# *In silico* modelling of organ-on-a-chip devices: an overview

**DOI:** 10.3389/fbioe.2024.1520795

**Published:** 2025-01-27

**Authors:** Yue Wang, Lucia Marucci, Martin E. Homer

**Affiliations:** ^1^ School of Engineering Mathematics and Technology, University of Bristol, Bristol, United Kingdom; ^2^ School of Cellular and Molecular Medicine, University of Bristol, Bristol, United Kingdom; ^3^ Bristol BioDesign Institute, University of Bristol, Bristol, United Kingdom

**Keywords:** organ-on-a-chip, organoids, computational modelling, mathematical modelling, microfluidic dynamics

## Abstract

An organ-on-a-chip (OOAC) is a microscale device designed to mimic the functions and complexity of *in vivo* human physiology. Different from traditional culture systems, OOACs are capable of replicating the biochemical microenvironment, tissue-tissue interactions, and mechanical dynamics of organs thanks to the precise control offered by microfluidic technology. Diverse OOAC devices specific to different organs have been proposed for experimental research and applications such as disease modelling, personalized medicine and drug screening. Previous studies have demonstrated that the mathematical modelling of OOAC can facilitate the optimization of chips’ microenvironments, serving as an essential tool to design and improve microdevices which allow reproducible growth of cell culture, reducing the time and cost of experimental testing. Here, we review recent modelling approaches for various OOAC devices, categorized according to the type of organs. We discuss the opportunities for integrating multiphysics with multicellular computational models to better characterize and predict cell culture dynamics. Additionally, we explore how developing more detailed OOAC models would support a more rapid and effective development of microdevices, and the design of robust protocols to grow and control cell cultures.

## 1 Introduction

Traditional 2D cell culture lacks the capability to reconstruct complex *in vivo* conditions, and so animals have been extensively used in research for physiological and disease studies, as well as for drug testing. However, animal research is expensive, time-consuming, and can raise ethical concerns (Huh et al., 2011). Moreover, the results derived from animal tests are often not directly applicable to understanding human physiology or predicting human physiological responses to diseases, drugs, or other stimuli. The 3Rs (Replacement, Reduction, Refinement) framework aims to reduce the number of animals used in experiments or to replace animals with alternative methodologies (Russell and Russell, 1957). Organ-on-a-chip (OOAC) technologies are considered a valuable alternative tool (Huh et al., 2012).

Unlike conventional static culture systems, OOACs allow to culture cells in a dynamic environment. By exerting control over fluid flow and the supply of nutrients or biochemical signals to the cells, the fluid shear force exerted on the cultures and concentration gradients can be tuned to provide spatiotemporal cues for specific model organs. Meanwhile, mechanical stresses to represent *in vivo* effects such as intestinal peristaltic motion or lung breathing can also be applied to the culture (Huh et al., 2011). To date, OOAC for various organs, including the lung (Huh et al., 2010), intestine (Kim et al., 2012), liver (Prodanov et al., 2016) and kidney (Jang et al., 2013), have been developed. However, this advanced technique comes with limitations. As many conditions governing organogenesis remain unknown, the culture of cells or organs can exhibit high variability and low repeatability (Park et al., 2019). Moreover, designing and developing the function of a microfluidic platform via traditional lab experiments can be costly and time-consuming (Sheidaei et al., 2020). Thus, mathematical modelling could be considered a valuable tool to help understand the microenvironment conditions inside the microfluidic devices and improve the culture systems.

Here we focus on OOAC devices developed for three organ types: lung (Francis et al., 2022; Kim et al., 2023), intestine (Zhang and Qiao, 2023), and liver (Dalsbecker et al., 2022), and summarise the current state-of-the-art in their corresponding *in silico* simulations. We also discuss the opportunities and challenges of integrating *in silico* models of OOAC devices with multicellular computational models. By utilizing the integrated model to predict culture conditions inside the chip, we may better characterize the physical and chemical cues for culturing cells and improve OOAC devices.

## 2 Organ-on-a-chip

To reproduce the physiological environment of the human body in the OOAC design, three main aspects should be considered: the fluid shear force which influences organ polarity (Theobald et al., 2018), the concentration gradient of biochemical signals present in physiological processes (Berthier et al., 2014), and the mechanical stress reflecting pressures in organs (Wu et al., 2020). To achieve precise control of biochemical molecules, an OOAC device is designed on a microscale to establish stable and controllable laminar flow. Since mixing among laminar flows is solely due to diffusion, biochemical gradients are predictable if we know the material properties of the OOAC fluid (e.g., density and viscosity) and operational parameters such as the fluid velocity, the travel distance of the monitored molecules, and the hydraulic diameter within the chip (Ferry et al., 2011). By tuning these parameters and designing a proper OOAC geometry, the concentration of the biochemical gradient can be controlled spatially and temporally, thereby more accurately simulating signal transport in the human body. Controlling the fluid flow in OOAC devices can also enable the generation of fluid shear forces within the culture environment, while aspects of the physical environment, such as peristaltic motions in the intestine or the respiration movements over the diaphragm, are typically modelled by stretching an elastic porous membrane between the culture chambers inside the chip. Different organ types have various requirements for chip design. In the following sections, the most recent *in silico* models of OOAC in the lung, intestine and liver will be introduced, respectively. Table 1 summarises the key features of these models and the OOAC technologies on which they are based.

**TABLE 1:** A summary of recent organ-on-a-chip (of lung, intestine, liver) mathematical models.

Organ	Devices features	Simulation software	Cell lines	Drug	Shear force	Gradient	Details	Ref.
Lung	PDMS; Double layer; Porous membrane; mechanical stress	COMSOL	N/A	N/A	✓	✓	Demonstrated the complex fluidic dynamics of the chip. Can estimate the transport of diluted species across the devices	Hancock and Elabbasi, (2015)
		COMSOL	N/A	N/A	✓	✓	Introduced an effective elastic modulus of the porous membrane into the simulation	Hancock and Elabbasi, (2016)
		COMSOL	N/A	N/A	✓	✓	Simulate the transport and absorption of nanoparticles under various breathing patterns within the device	Amin Arefi et al. (2020)
Intestine	PDMS; Double layer; PTFE membrane	COMSOL	Colorectal cancer cells	Oxaliplatin	✓	✓	Can test the drug Oxaliplatin on cancer cells. Can predict the drug concentration in the device overtime. Can be used for optimizing the dosing strategies	Komen et al. (2020)
Liver	PDMS, PMP, COC; Double layer	COMSOL	Hepatocyte, endothelial cell	N/A	✗	✓	Can estimate the oxygen levels for various cell types in devices. Three materials were tested, PMP was suggested as a promising material	Ochs et al. (2014)
	PDMS; Double layer; Liver-lobule-like chambers	COMSOL	Human hepatocellular carcinoma cells, hiPSC-derived hepatocytes	N/A	✓	✓	A very large-scale liver-lobule-on-a-chip. Fluidic dynamics and the diffusion of glucose in microchannels were simulated	Banaeiyan et al. (2017)
	PDMS; Double layer; Porous membrane; mechanical stress	GAMBIT, FLUENT	Kupffer cell, stellate cell, hepatocyte, sinusoidal endothelial cell	N/A	✓	✗	Computational simulation was utilized to analyze the flow field and mass transfer inside the device	Du et al. (2017)
		MATLAB	N/A	N/A	✓	✓	Immersed Boundary Methods simulations were constructed. Flow flux, wall shear stress, transmembrane pressure difference were confirmed to be dependent on chip geometry and membrane permeability	Chen et al. (2020)

### 2.1 Lung

As part of the respiratory system, human lungs are responsible for exchanging air with the blood to facilitate human breathing. During the process of oxygen and carbon dioxide exchange, the lungs are exposed to a risky environment and can suffer from diseases such as cancer, asthma, and influenza. To study lung conditions and respiratory diseases, preclinical models are often used, although they can be accompanied by many limitations. In response to these challenges, lung-on-a-chip (LOAC) devices are a promising new approach. The lung is a complex organ with tree-like airways extending from the trachea and ending with millions of alveoli where air exchange occurs. To mimic the *in vivo* microarchitecture and mechanical conditions of the lung, tissue-tissue interactions, air-liquid interfaces and mechanical stretching due to breathing must be reconstituted (De et al., 2022).

**FIGURE 1:**
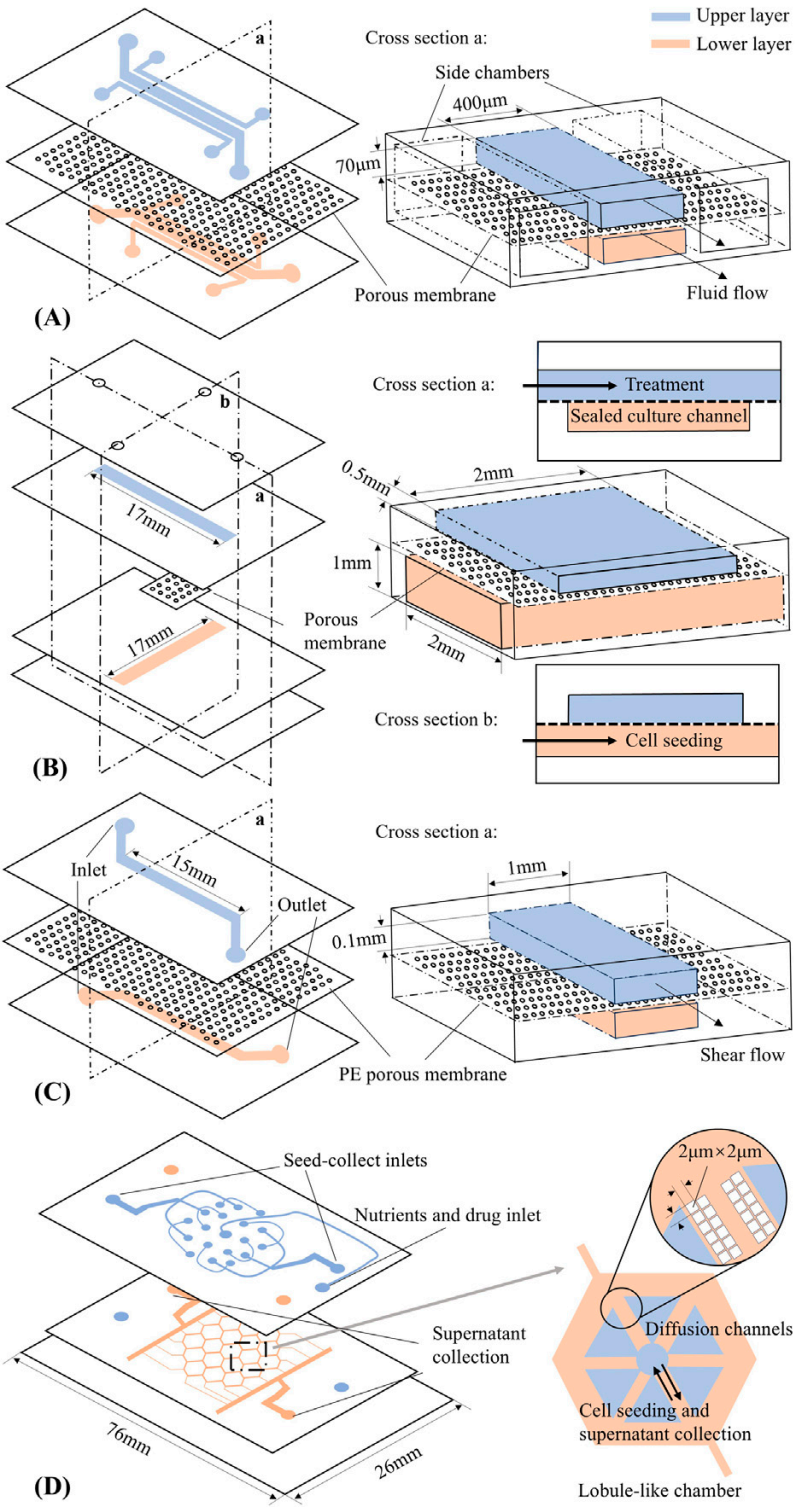
Organ-on-a-chip schematic designs (not to scale). Upper and lower layer channels are shown in blue and pink, respectively. **(A)** Lung-on-a-chip (Huh et al., 2010). **(B)** Intestine-on-a-chip (Komen et al., 2020). **(C)** Liver-on-a-chip (Du et al., 2017). **(D)** Liver-lobule-on-a-chip (Banaeiyan et al., 2017).

Huh et al. (2010) implemented the first LOAC using a human lung alveolar epithelial cell line. This development successfully replicated organ-level physiological and pathophysiological responses on a chip. As shown in Figure 1A, the lung chip consists of two parallel microchannels separated by a thin polydimethylsiloxane (PDMS) porous membrane coated with extracellular matrix (ECM). Human alveolar epithelial cells were cultured in the upper air channel, where air could be introduced, while human pulmonary microvascular endothelial cells were cultured in the bottom blood channel to achieve an alveolar-capillary interface. Two chambers beside the double-layer culture area were designed to produce elastic deformation of the microchannels, thus performing mechanical stretching in the microsystem to replicate breathing movements.

An *in silico* model of Huh et al.’s microdevice was developed by Hancock and Elabbasi (2015) using COMSOL Multiphysics^®^. The model configured the architecture, materials, laminar fluid flow and applied mechanical forces within the chip based on parameters from previous literature (Huh et al., 2010) (see the schematic shown in Figure 1A) and analysed the fluidic dynamics across the device. Computational simulations provided estimates of the transport of diluted species, such as drugs and nutrients, across the membrane and computed the shear stress on cells. Subsequently, Hancock and Elabbasi (2016) further advanced this work by introducing an effective elastic modulus of the porous membrane into the previous model. The enhanced model could be used to compute the effective molecular diffusivity and effective thermal diffusivity near the porous membrane. These factors influence heat and mass transfer through the air-liquid interface of the device, and their impact on cell cultures such as shear stress and biochemical gradients are worthy of further study. However, neither model explicitly includes the multicellular structure that represents the organ in the LOAC device, and so its growth and development, and how that may depend on the device design must still be studied experimentally.

One application of the LOAC is to model the transport of nanoparticles (NPs) in the lung. Nanoparticles range in size from 1 to 100 nm and can originate from sources such as cigarette smoke, air pollution, or viruses. In the post-COVID-19 pandemic period, respiratory-related diseases have become a concern, and clinical research on NPs has attracted increased attention. In the pursuit of more effective testing of NPs, Amin Arefi et al. (2020) proposed an *in silico* model to study the transport and absorption of NPs within a LOAC. The simulation was based on the design of Huh et al.‘s LOAC (Figure 1A) and aimed to estimate NP transport under various breathing patterns and to investigate the potential effects that different NP sizes may introduce. Using COMSOL to solve the Navier-Stokes equations for fluid flow in the chip, and employing Eulerian advection-diffusion, Lagrangian particle tracking for fine and coarse NP tracking, and Langmuir and Frumkin kinetics for computing the surface adsorption and desorption, the results indicated that particle deposition increases during breathing with high frequency or long breath-holding time. Additionally, the findings highlighted that both particle sizes and the orientation of the lung epithelium layer relative to gravity affect deposition efficiency.

### 2.2 Intestine

The human intestine is responsible for a range of digestion, absorption, and secretion processes. Serving as a barrier between the intestinal contents and other parts of the human body, it plays a crucial role in oral drug absorption. Meanwhile, physiologically, the intestine hosts a rich microbiome and communicates with other organs to regulate body function. To model these properties, dynamic mechanical stress, precise signal transport, and co-culture ability are required to be replicated in a model culture system (Bein et al., 2018).

To date, one of the most commonly used devices in experimental work related to gut-on-a-chip (GOAC) is the chip designed by Emulate Inc., initially developed and published by Huh et al. (2010). Kim et al. (2012) first applied the microfluidic chip technique for GOAC, subesequently developed by Kim and Ingber (2013). With a design similar to the device described earlier in section 2.1 (Figure 1A), this chip also features two parallel channels separated by an ECM-coated porous membrane, allowing observation of diffusion between two channels. PDMS was used as the material for both the chip and membrane. In this configuration, human Caco-2 intestinal epithelial cells (Caco-2 human colorectal carcinoma line (Peterson and Mooseker, 1992)) were cultured in the upper channel, while the culture medium was perfused through both channels at the same flow rate. The epithelial monolayer forms on the porous membrane, and the peristaltic motions of the intestine epithelium are mimicked by the tractions applied by the two side chambers to the membrane in between. Specifically, the formation of finger-like structures called intestinal villi, lined by polarized epithelial cells and separated by crypts, was observed. Furthermore, Kasendra et al. (2018) presented additional studies of GOAC using human intestinal epithelial cells derived from organoids and microvascular endothelial cells. In this work, organoid cells grew into epithelium with finger-like structures, closely mimicking the structure of the human duodenum *in vivo* and also performing the intestinal barrier functions.

Komen et al. (2020) designed a different GOAC with the specific aim of exposing cancer cells to the drug oxaliplatin through dynamic perfusion. The chip design is illustrated in Figure 1B, featuring two crossing channels. The upper channel is for drug dosing and the sealed lower channel is for seeding cells. The cell culture takes place in the intersection of the two channels. *In silico* modelling with COMSOL Multiphysics^®^ in two space dimensions was used to predict the drug concentration for the lower channel over time. Within the simulation, the two-dimensional diffusion equation was solved for the flux and concentration of the solute according to Fick’s law. An optimal value of membrane porosity was calculated and used in subsequent experiments to design the thickness and pore size of the membrane.

### 2.3 Liver

The liver is a vital metabolic organ with many essential functions, including protein synthesis and organism detoxification. A liver chip is typically expected to replicate the architectural features, metabolic capabilities, regenerative properties, and injury response of a living liver. Within the liver, the liver sinusoids play a crucial role in regulating blood flow and thus become a key component to be reconstructed on a liver chip. A typical liver sinusoidal chip may consist of four main types of hepatic cells: liver sinusoidal endothelial cells, Kupffer cells, hepatic stellate cells, and hepatocytes within two fluid channels, i.e., sinusoidal vascular channel and Disse space (Deguchi and Takayama, 2022).

A microfluidic system mimicking the liver sinusoidal structure was developed by Du et al. (2017) (Figure 1C). Similar to the chip designed by Huh et al. (2010) (Figure 1A), the liver chip consists of two PDMS layers separated by a porous polyester membrane, with Collagen I pre-coated on both the channels and the membrane. The Kupffer cells and liver sinusoidal endothelial cells are cultured on the membrane, while the hepatic stellate cells and hepatocytes are cultured in the lower channel. As a result, the fundamental architecture of liver sinusoids was replicated, an *in vivo* monolayer of liver sinusoidal endothelial cells was successfully established, and metabolic activity was activated through the co-culture of hepatocytes and nonparenchymal cells. Du et al. (2017) also constructed an *in silico* model: a computational fluid dynamics simulation of the chip geometry was constructed in FLUENT software, and particle tracking visualization tests were used to estimate the flow field inside the chip at different heights. The computational results were validated against experimental data. By configuring the parameters of the chip geometry and considering the velocity of the laminar flow through the system and viscosity of the fluid, the fluid flow and mass transfer inside the chip could be better understood.

Another advanced liver chip design is the very large-scale liver-lobule (VLSLL)-on-a-chip proposed by Banaeiyan et al. (2017) (Figure 1D). The two-layer PDMS device consists of multiple hexagonal culture chambers that mimic the natural geometry of liver lobule: a combination of the hepatic lobule structure and the blood-circulation network. Each chamber has a bottom layer for tissue culture and a top layer for the seed-feed network, connected via a central opening that mimics the central vein. This design helps protect the cultured cells from the high shear stress of fluid flow. Unlike typical designs, this system relies on diffusion-convection effect for nutrient delivery, and it is validated through *in silico* models. Fluid dynamics within the device were analyzed to optimize key design parameters, such as flow velocity and shear stress. The model also explored glucose diffusion across the chambers, confirming that diffusion continues until the glucose concentration in the center of the chamber reaches a steady state. These simulations were crucial for predicting nutrient transport within the chip and optimization of the device. As a result, the chip provides a robust platform for studying liver function and high-throughput drug metabolism. These functionalities were experimentally validated by culturing human hepatocellular carcinoma cells and human-induced pluripotent stem cell-derived hepatocytes separately on the proposed device.

Subsequent work by Chen et al. (2020), based on (Du et al., 2017), further demonstrated that *in silico* flow field simulation could enhance chip design. An existing problem in the chip design is that chip geometry parameters such as pore size, channel size, membrane thickness, and membrane permeability all have a significant influence on the flow features inside the device and can affect the culture functions. A systematic analysis is required in this case to quantify their relationship and optimize the design. Chen et al. (2020) used immersed boundary methods to construct the *in silico* model framework in MATLAB, with theoretical modelling supporting assumptions of the immersed boundary method model. The simulations investigated the dependence of fluid dynamics on chip geometry (height, width), membrane permeability, and fluid viscosity. The results showed that increasing the lower channel height initially increased and then decreased the wall shear stress. It was also found that increasing the channel length or membrane permeability enlarged the transition threshold of the maximum wall shear stress. Moreover, the transmembrane pressure difference and wall shear stress increased with increasing viscosity. Chen et al. (2020) suggest that this may indicate hepatocytes could experience higher wall shear stress as blood viscosity increases, although their model does not explicitly include the cellular structures typically found in the liver.

*In silico* models have also been used to aid in predicting the oxygen level in a liver chip. To understand cell culture with hypoxic or anoxic conditions, and examine cell viability and behaviour under specific oxygen-levels, thermoplastic devices made from oxygen-impermeable materials are considered as an alternative to PDMS, Ochs et al. (2014) developed a computational model to predict oxygen levels in devices made of oxygen-permeable materials PDMS and poly (methyl pentene), and the oxygen-impermeable material cyclic olefin copolymer during cell culture. Assuming that endothelial cells and hepatocytes were cultured on the chip with a known oxygen uptake rate, Ochs et al. (2014) estimated cell respiration in devices; computational results were validated experimentally. Their results verified that moderate media flow rates or an oxygen-permeable film can help counteract oxygen depletion in non-permeable devices. Finally, poly (methyl pentene) was introduced as a suitable material due to its good oxygen permeability and property of maintaining sufficient oxygen levels for high metabolism cell types.

## 3 Discussion

In the previous section, we described a range of OOAC devices that have been designed and developed to reproduce different organ characteristics, and the associated *in silico* modelling that has proven to be a reliable and efficient tool to assist in chip design. By optimizing parameters such as chip geometry and flow characteristics within the devices, shear forces and mass transfer exerted on the cultures can be adjusted to achieve precise control over the microenvironment in the devices. However, challenges still remain. In particular, the complexity of biological systems, including cellular behavior and cell-matrix interactions, is not yet fully understood. Most of the simulation frameworks do not consider the dynamics and characteristics of cells and organs cultured on the chip; this makes it difficult to determine which parameters (such as flow rate, oxygen concentration, nutrient concentration, etc.) should be appropriate for each type of OOAC culture. Typically, laboratory experiments are needed to draw such conclusions. We believe that developing *in silico* modelling frameworks that combine multicellular behavior with microfluidic analysis has the potential to better assist in understanding the growth of cells within these devices, designing appropriate chips with corresponding parameters, and ensuring reproducibility across experiments.

A multicellular model typically refers to a computational model that can recapitulate multiscale temporal processes of cells and tissues such as shape changes, movement, division and apoptosis, as well as intracellular processes such as signalling pathway dynamics, and responses to endogeneous and exogenous stimuli. Different approaches have been implemented using various computational techniques including agent-based modelling, continuum modelling, and differential equations (Fletcher and Osborne, 2022). A comprehensive review of recent developments in multiscale modelling to recapitulate organoids dynamics was provided by Montes-Olivas et al. (2019).

However, to the best of our knowledge, there is currently no integrated modelling framework that accounts for both cellular processes and fluid dynamics in OOAC devices. There are studies that integrate fluid dynamics with cellular behaviour, as a classical fluid-structure interaction problem. For example, Polwaththe-Gallage et al. (2014) describe a methodology to implement the 3D geometry of red blood cells (RBCs) in COMSOL Multiphysics^®^. In this work, the red blood cell membrane geometry is initially generated as a spherical mesh. Particles with finite masses are positioned on each mesh node, and an elastic spring connects each particle to its six neighbors. External forces applied to specific particles cause changes in spring length, refreshing the coordination of each particle and presenting in-plane deformation in the red blood cell geometry. Forces due to spring-generated deformations, bending deformation, surface and volume constraint energies are implemented. Ni et al. (2015) model red blood cell motion and deformation through a microchannel filled with an alcohol plasma solution, implementing a 2D single red blood cell model in COMSOL Multiphysics^®^. The red blood cell model includes a 2D deformable contour, and membrane interaction with fluid is implemented using an elastodynamic equation, while the surrounding medium is modelled as an incompressible Newtonian fluid with the COMSOL Multiphysics^®^ built-in Navier-Stokes solver. The results showed that, with the same velocity, the deformability of red blood cells would increase with increasing viscosity. Such red blood cell studies demonstrate the capability of COMSOL Multiphysics^®^ to simulate a single cell’s deformation due to its surrounding environment. However, simulating organogenesis in COMSOL Multiphysics^®^ presents challenges in modelling the cells with their intrinsic mechanics and proliferative abilities. Peters and Iber (2017) addressed organogenesis simulation in COMSOL Multiphysics^®^, but focused on the tissue viscoelastic behavior, similar to red blood cell models.

The multicellular processes involved in organogenesis, in the absence of a surrounding fluidic environment, are more usually studied in specialised agent-based modelling and simulation environments such as CHASTE (Osborne et al., 2017), CompuCell3D (Swat et al., 2012), PhysiCell (Ghaffarizadeh et al., 2018), and Morpheus (Starruß et al., 2014). One notable example of coupling cell-based models with cell-extracellular interactions is the work of Osborne and Bernabeu (2018). Building on a previous study of artery remodelling (Bernabeu et al., 2013), by combining the CHASTE framework (Pitt-Francis et al., 2009) with HemeLB (Mazzeo and Coveney, 2008) (a lattice-Boltzmann solver for simulating blood flow), they illustrate vascular tissue mechanics under hemodynamics. The relaxation and expansion of the vessel wall due to varying flow speeds are observed. While this work demonstrates effective practice in combining cell-based models with fluid forces, direct application of the CHASTE-HemeLB coupling to OOAC simulations still poses challenges. HemeLB is specifically designed for modelling blood flow in vessel geometry, and its geometry dataset is fixed, generated using HemeLB’s built-in graphical editing tool or obtained from X-ray or Magnetic Resonance Angiography scans. Therefore, bridging this gap requires either integrating multicellular simulation with microfluidic aspects into existing cell-based models with commercial software, or implementing a fluid dynamics module in biological-based model frameworks.

To conclude, OOAC is a technology with huge potential for understanding human physiology with applications such as disease modelling and drug testing. OOACs for a variety of organs have been developed to recapitulate the essential functions of their corresponding organs. *In silico* models have been implemented to investigate the shear stress, mass transfer, and diffusions via the air-liquid and/or liquid-liquid interface of these devices. By predicting the dynamic microenvironment in advance, chip design could be enhanced and optimized, thereby reducing the time and cost in practical experiments. However, cell-environment interactions are rarely considered in detail in these *in silico* models, and it would be worthwhile to develop integrated models including both chip dynamics and multicellular interactions.
